# Systemic *Mycobacterium kansasii* Infection in Two Related Cats

**DOI:** 10.3390/pathogens9110959

**Published:** 2020-11-18

**Authors:** Petra Černá, Jordan L. Mitchell, Joanna Lodzinska, Paola Cazzini, Katarina Varjonen, Danièlle A. Gunn-Moore

**Affiliations:** 1 Department of Clinical Sciences, Colorado State University, Fort Collins, Colorado, CO 80528, USA; petra.cerna@colostate.edu; 2 The University of Veterinary and Pharmaceutical Sciences Brno, 612 42 Brno, Czech Republic; 3 The Royal (Dick) School of Veterinary Studies and The Roslin Institute, Easter Bush Campus, University of Edinburgh, Midlothian EH25 9RG, UK; jlodzins@exseed.ed.ac.uk (J.L.); Paola.Cazzini@ed.ac.uk (P.C.); Danielle.Gunn-Moore@ed.ac.uk (D.A.G.-M.); 4 AniCura Djursjukhuset Albano, Rinkebyvägen 21A, 182 36 Danderyd, Sweden; katarina.varjonen@anicura.se

**Keywords:** feline cutaneous mycobacteriosis, non-tuberculous mycobacteria, *Mycobacterium kansasii*, diagnostics, treatment

## Abstract

Mycobacterial infections are a major concern in veterinary medicine because of the difficulty achieving an etiological diagnosis, the challenges and concerns of treatment, and the potential zoonotic risk. *Mycobacterium kansasii*, a slow-growing non-tuberculous mycobacteria, causes disease in both humans and animals. While infections have been well described in humans, where it may be misdiagnosed as tuberculosis, there are fewer reports in animals. Only four cases have been reported in the domestic cat. This case report describes systemic *M. kansasii* infection in two sibling indoor-only cats that presented two and half years apart with cutaneous disease that was found to be associated with osteolytic and pulmonary pathology. Infection with *M. kansasii* was confirmed in both cats by polymerase chain reaction on fine-needle aspirate of a lumbosacral soft tissue mass in one cat and on a tissue punch biopsy of a skin lesion in the other; interferon-gamma release assay inferred *M. avium*-complex and *M. tuberculosis*-complex infection in the two cats, respectively. Both patients made a full recovery following antimicrobial therapy with rifampicin, azithromycin, and pradofloxacin (plus N-acetyl cysteine in cat 2). This report highlights successful treatment of systemic *M. kansasii* mycobacteriosis in the cat and the challenge of accurately diagnosing this infection.

## 1. Introduction

Mycobacterial infections in cats are increasingly recognised throughout many parts of the world, including Great Britain [1], continental Europe [2,3,4,5], Australia [6], and North America [7]. Broadly speaking, mycobacteria can be divided into two groups: the non-tuberculous mycobacteria (NTM) [8], and the *Mycobacterium* (*M.*) *tuberculosis*-complex (MTBC), which includes the causative agents of tuberculosis (TB) in cats, i.e., *M. bovis* and *M. microti*—the vole bacillus [1,9]. There are over 150 NTM species [10], many of which are important pathogens in their own right e.g., *M. avium* subsp. *paratuberculosis*, the causative agent of Johne’s disease in cattle and sheep [11], while others are opportunistic environmental saprophytes [12]. Historically, the NTM have been categorized based on their growth characteristics [13]; however, some species of mycobacteria cannot be cultivated in standard laboratory settings, such as *M. leprae*, which causes leprosy (Hansen’s Disease) in humans [14], and its murine equivalent *M. lepraemurium*, which has been attributed as causing ‘feline leprosy syndrome’ [15]. With the advancement of molecular testing, there has been a trend towards classifying NTM based upon genetic similarity rather than phenotypic and growth characteristics; this has also allowed for the identification of previously unrecognized mycobacterial species [16,17].

*Mycobacterium kansasii*, a slow-growing NTM [18], was first isolated in 1953 [19]. Since then, it has been identified as a major pathogen of people, where it can cause pulmonary disease resembling TB [20,21]. Cutaneous lesions may also develop [22], and co-infection with human immunodeficiency virus is frequent [20]. Infections with NTM in domestic and wildlife species, including *M. kansasii*, have been reviewed previously [23]; in the United States, *M. kansasii* comprised nearly 2% of culture-positive samples from cattle submitted for national bovine TB testing [24]. Six subtypes of *M. kansasii* have been identified, although it has been proposed that these should be elevated to species-level recognition [25]. While *M. kansasii* has been identified as causing disease in a number of different animal species [23,24,26,27,28,29,30,31], to the authors’ knowledge, it has only been reported four times in the domestic cat; treatment with doxycycline was unsuccessful in one case [32], but a second cat was successfully treated with clarithromycin and rifampicin [33]. Clinical outcomes were not reported in the remaining two cases [34].

There is significant overlap in the clinical presentation of feline mycobacteriosis, regardless of which species of mycobacteria is involved [35], which can make obtaining a definitive etiological diagnosis challenging. Hematological and serum biochemistry changes, if present, are non-specific and vary with the severity of disease. Hypercalcemia may be identified and appears to correlate with extensive or disseminated granulomatous disease [1], while the serum concentration of active vitamin D appears to be lower in cats with mycobacterial disease [36]. Unlike in humans, co-infection of mycobacteria and immunodeficiency viruses—in cats, feline leukemia virus (FeLV) and feline immunodeficiency virus (FIV)—are rare [1]. Cytological and histological samples show granulomatous to pyogranulomatous inflammation, predominated by macrophages, which may be foamy in appearance, and necrosis may also be present [37]. Variable numbers of negative-staining, linear to comma-shaped microorganisms, can be seen within macrophages or in the background of cytological preparations [38]. Aspirates and biopsy samples should always be Ziehl-Neelsen (ZN) stained to confirm the presence of acid-fast bacilli (AFB). In those cases where there is a suspicion of mycobacterial infection, but rare or no microorganisms can be identified with routine staining, ZN staining greatly enhances the possibility of identifying AFB. However, approximately two-thirds of biopsy specimens with histopathological changes indicative of mycobacterial infection are negative on ZN staining for AFB [39]. Therefore, a negative ZN stain cannot be used to rule out mycobacteriosis. In the UK, the reference standard diagnostic test for companion animal mycobacterial disease is specialist culture; however, approximately half of the samples fail to grow [1], and it can take longer than three months for colonies to be identified with some mycobacterial species, such as *M. microti* [40]. More rapid diagnostic tests are available; namely, polymerase chain reaction (PCR), which can identify mycobacterial DNA and may be able to provide speciation [7,41,42,43]; however, they are often expensive and have poor sensitivity on formalin-fixed, paraffin embedded tissues compared to fresh samples [44]. The interferon-gamma (IFNγ) release assay (IGRA) [45,46], adapted for use in cats from cattle and humans [47,48], is a highly sensitive immunological test for MTBC infections, although it is limited in its ability to diagnose and discriminate between infections with NTM [49].

This case report presents two sibling cats from the same household that were diagnosed with *M. kansasii* infection. Both cats presented with cutaneous lesions, but diagnostic investigations revealed systemic mycobacteriosis including bone involvement with osteolysis. The IGRA results were discordant; *M. kansasii* infection was diagnosed by PCR, highlighting the limitations of immunological assays with this species of mycobacterium and the potential implications for misdiagnosis. Antimycobacterial therapy was undertaken and remission of clinical signs was achieved in both cats. Curiously, the second cat developed overt disease two and a half years after the first cat presented with cutaneous lesions. 

## 2. Results

### 2.1. Cat 1

**Figure 1:**
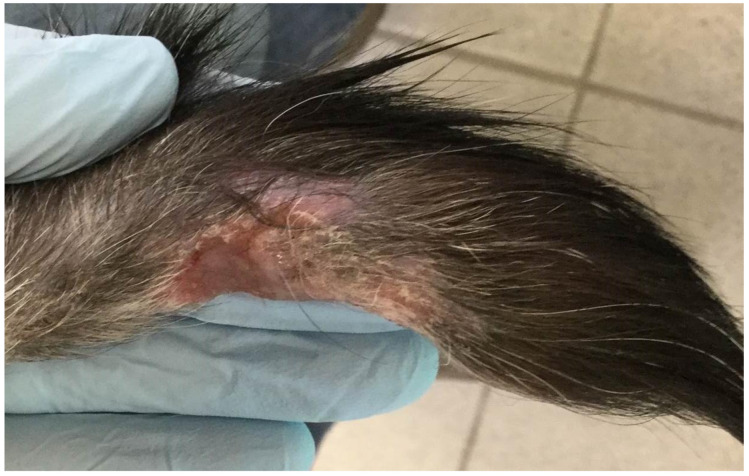
Non-healing lesion on the tail of cat 1 believed to be self-inflicted trauma but mycobacterial infection could not be ruled out.

An eight-year-old male neutered Domestic Shorthair (DSH) cat presented in February 2017 to the Hospital for Small Animals, The Royal (Dick) School of Veterinary Studies (RDSVS), The University of Edinburgh, for investigation of over-grooming of the distal tail and an associated chronic non-resolving cutaneous lesion caused by self-inflicted trauma; partial amputation by the referring veterinarian surgeon (RVS) to remove the affected part of the tail had not resolved the chewing, and resulted in the cat starting to chew a new area of the tail. The patient was born in Italy in a multi-cat household and had an outdoor, feral lifestyle from which they were rescued, then lived for a period of time in Philadelphia, PA, USA, and in New York, NY, USA, prior to moving to Scotland, UK in March 2014. A sibling female cat (cat 2) was kept in the same household and was clinically healthy. Both cats were indoor only and were fed a commercial dry cat food diet. On initial physical examination the patient was bright, alert, and responsive. Physical examination identified a grade III/VI heart murmur and an ulcerated, alopecic lesion on the underside of the tail (Figure 1), believed to be a result of self-trauma. Retinal examination showed changes suggestive of historical taurine deficiency. The remaining physical examination was unremarkable. Cytology from the surface of the tail lesion showed neutrophils and eosinophils, but no evidence of infection. An echocardiogram was performed; an intermittent right ventricular outflow tract obstruction was believed to be the cause of the auscultated murmur. Neurological examination revealed pain on deep palpation of the lumbosacral region but was otherwise unremarkable. Radiographs of the pelvis and tail showed mild narrowing of the intervertebral disc space between L7-S1, mild subluxation of the seventh caudal vertebra, and a soft tissue opacity ventral to the sixth and seventh caudal vertebrae that was thought to be either congenital or traumatic in origin. Treatment with gabapentin (10 mg/kg PO q24 h; Bova) and meloxicam (0.05 mg/kg PO q24 h; Metacam, Boehringer Ingelheim) were initiated for suspected neuropathic pain causing tail chewing, and chlorhexidine gluconate (q12 h; HibiScrub, Mölnlycke) was applied to the tail lesion to address any potential secondary infection that could develop, which was continued by the owners at home.

**Figure 2:**
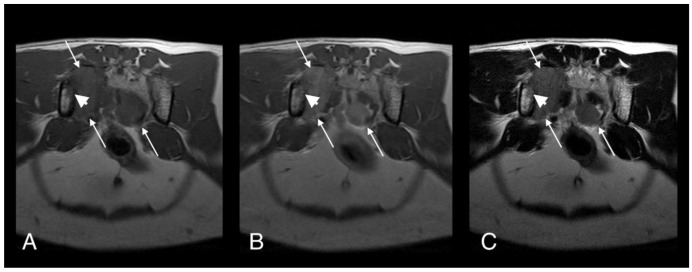
T1-weighted (**A**), T1-weighted after gadolinium contrast medium administration (**B**), and T2-weighted (**C**). Magnetic resonance imaging (MRI) images of cat 1 in transverse plane at the level of the sacrum. Note the heterogeneous and contrast enhancing soft tissue mass originating at the level of the right sacroiliac joint (long arrows). The mass is causing osteolysis of the right sacroiliac joint with bone defect where the normal hypointense bone margin is no longer present (short arrows).

**Figure 3:**
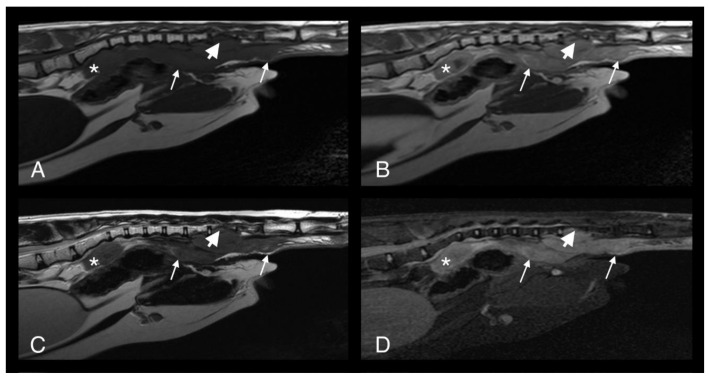
T1-weighted (**A**), T1-weighted after gadolinium contrast medium administration (**B**), T2-weighted (**C**) and short-tau inversion recovery (STIR) (**D**) MRI midline images of cat 1 in the sagittal plane at the level of the caudal abdomen and tail. Note the heterogeneous and contrast enhancing soft tissue mass originating at the ventral aspect of the tail base (long arrows). The mass is causing osteolysis of the haemal arches of the caudal vertebral bodies (short arrows) where the normal hypointense bone margin is no longer present. The sacral mass is also visible (asterisk).

**Figure 4:**
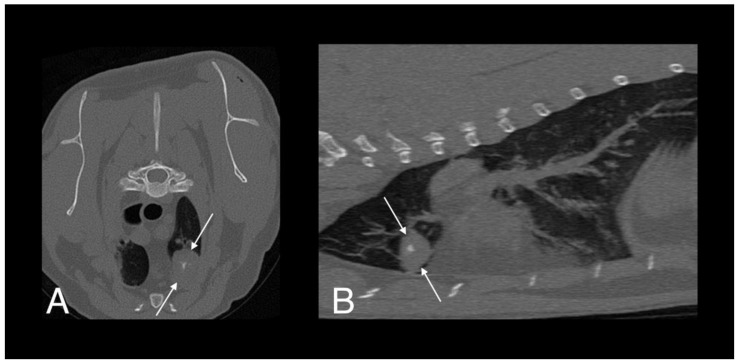
Computed tomography (CT) images of cat 1 in transverse (**A**) and sagittal (**B**) planes showing a well-defined pulmonary mass with central mineralization (arrows).

The patient re-presented one month later for continued tail chewing and no improvement on gabapentin and meloxicam. Hematology was unremarkable, while creatinine (270 umol/L; reference interval [R1] 40–177) and urea (23.6 mmol/L; RI 2.8–9.8) were increased on serum biochemical analysis. Magnetic resonance imaging (MRI) of the lumbosacral spine and caudal vertebrae demonstrated a large, expansile, heterogeneous, and strongly contrast-enhancing soft tissue lesion originating from the right sacroiliac joint. The mass was extending into the psoas musculature and causing osteolysis of the sacrum (Figure 2). A second heterogeneous soft tissue mass was identified in the ventral aspect of the tail base. This lesion was invading the hypaxial coccygeal muscles and causing mild osteolysis of the haemal arches of the coccygeal vertebrae (Figure 3). The regional lymph nodes were mildly enlarged. Whole body computed tomography (CT) was performed to further assess the osteolysis and for staging purposes. This confirmed the MRI findings affecting the tail and sacroiliac joint, but also found a large, round, partially mineralized soft tissue mass within the ventral portion of the left cranial lung lobe (Figure 4). 

**Figure 5:**
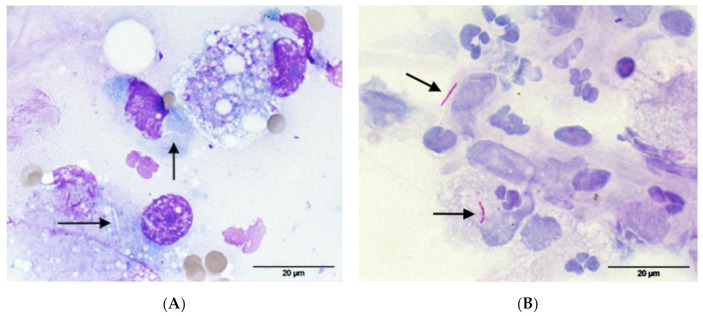
FNA from the soft tissue mass between the sacrum and rectum of cat 1, (**A**) May-Grünwald-Giemsa: notice the presence of negative-staining linear to comma-shaped microorganisms (arrows) within macrophages; (**B**) Ziehl-Neelsen staining: the microorganisms were positive for acid-fast staining confirming the suspicion of *Mycobacterium* species (arrows). Pictures were taken using a 100× oil objective for a total magnification of 1000×.

Based on the results of imaging, an aggressive neoplastic or infectious process was suspected. A fine-needle aspirate (FNA) sample was obtained via a para-rectal approach from the soft tissue mass between the sacrum and rectum, and was stained with routine May-Grünwald-Giemsa. The specimen had moderate cellularity; large foamy macrophages predominated, and they occasionally contained linear to comma-shaped, 1 × 5 microns, non-staining organisms compatible with *Mycobacterium* species (Figure 5A). A few bi- and multi-nucleated macrophages, moderate number of neutrophils, and occasional lymphocytes, plasma cells, and fibroblasts were also present. One of the slides was de-stained and re-stained with ZN, which confirmed the few microorganisms seen were acid-fast positive (Figure 5B). An IGRA was performed (Biobest Laboratories), showing a biased response to avian purified protein derivative (PPDA) over bovine purified protein derivative (PPDB), with no response to the antigenic cocktail of 6 kDa early secreted antigenic target (ESAT-6) and 10 kDa culture filtrate protein (CFP-10), which was interpreted as infection with a member of the *M. avium*-complex (MAC). A de-stained FNA slide was submitted for PCR (Leeds Teaching Hospitals NHS Trust) using a commercial kit (GenoType Mycobacterium; Hain Lifescience GmbH, Nehren, Germany) [50,51], which identified non-MTBC mycobacterial DNA; nucleotide sequence analysis of the 16S rRNA product gave a 100% similarity match for *M. kansasii*. 

Antimycobacterial treatment was initiated with rifampicin (15 mg/kg PO q24 h; Rifadin, Sanofi), azithromycin (16 mg/kg PO q24 h; Sandoz), and liquid pradofloxacin (6 mg/kg PO q24 h; Veraflox, Bayer). Buprenorphine (0.02 mg/kg PO q8–12 h; Buprecare, Animalcare) was prescribed to provide analgesia. Fusidic acid cream (q12 h; Fucidin, Leo) was applied twice daily after cleaning the tail lesion to address any secondary skin infection caused by the chewing. Supportive therapy with *S*-Adenosylmethionine (SAMe) (20 mg/kg PO q24 h; Hepaticare, then Hepatocyl (which contains additional silybin), Ceva) was initiated to reduce the potential hepatotoxic side effects of rifampicin. One month later, the patient’s tail lesion was almost healed. Hematology and serum biochemistry were performed to monitor for possible rifampicin or pradofloxacin toxicity; the results for both were unremarkable. The reason for the cause and then resolution of the previous azotemia was not identified. 

**Figure 6:**
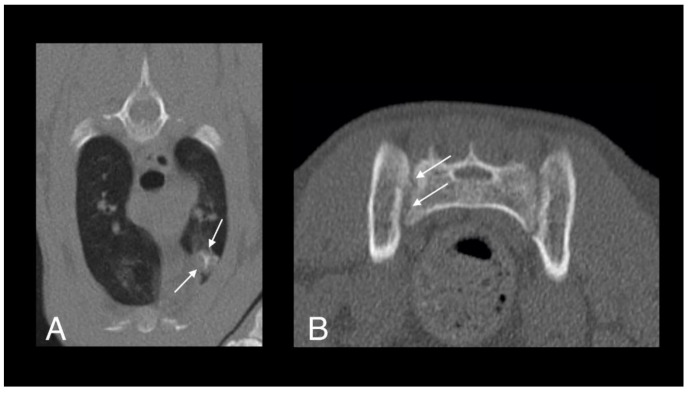
Follow up CT scan images of the lung (**A**) and sacroiliac joints (**B**) of cat 1, four months after starting antimycobacterial treatment. The lesion within the left cranial lung lobe is still visible but had moderately decreased in size (arrows). The osteolytic lesions of the right sacroiliac joint are subtle, with a mildly irregular margin of the joint surface (arrows). The soft tissue mass at the tail base is no longer present.

Four months later, a repeat CT scan was performed. The lesion within the left cranial lung lobe had decreased moderately in size (Figure 6A). The osteolytic lesions had almost completely healed, leaving only a mildly irregular margin of the right sacroiliac joint surface. The soft tissue mass at the tail base was no longer visible and the regional lymphadenopathy had resolved (Figure 6B). Hematology revealed leukopenia (5.2 × 10^9^/L; RI 7.0–20.0) with mild neutropenia (2.13 × 10^9^/L; RI 2.5–12.8). Serum biochemistry revealed mildly increased urea (16.9 mmol/L; RI 2.8–9.8) but was otherwise unremarkable. Echocardiographic changes were static from the previous findings five months prior.

A total of eight months after initiating antimicrobial therapy, a final CT was performed. The pulmonary nodule was static in size and appearance, there was no evidence of soft tissue mass recurrence and no new lesions were identified. Antimicrobial therapy was discontinued, and no clinical signs consistent with recrudescence or recurrence of infection have been observed during two and a half years of follow-up. 

### 2.2. Cat 2

**Figure 7:**
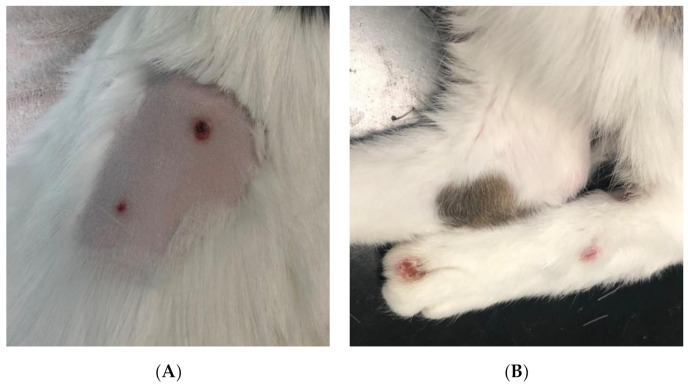
Circular, well circumscribed, ulcerative lesions on the dorsum (**A**) and distal pelvic limbs (**B**) of cat 2.

A ten-year-old female neutered DSH cat, sister of cat 1, presented in July 2019 to the RDSVS for investigation of a four-week history of coughing, and a small subcutaneous mass on the ventral thorax associated with the mammary tissue. The RVS had biopsied this mass, which was consistent with granulomatous inflammation; ZN staining for AFB was negative. An additional firm mass on the sternum was identified by the RVS on radiography. At the referral physical examination six weeks later, the patient was bright, alert, and responsive, with multiple ulcerative lesions on the caudal dorsum (Figure 7A) and distal to the hock on the pelvic limbs (Figure 7B). The rest of the physical examination was unremarkable, as were hematology and serum biochemistry. 

**Figure 8:**
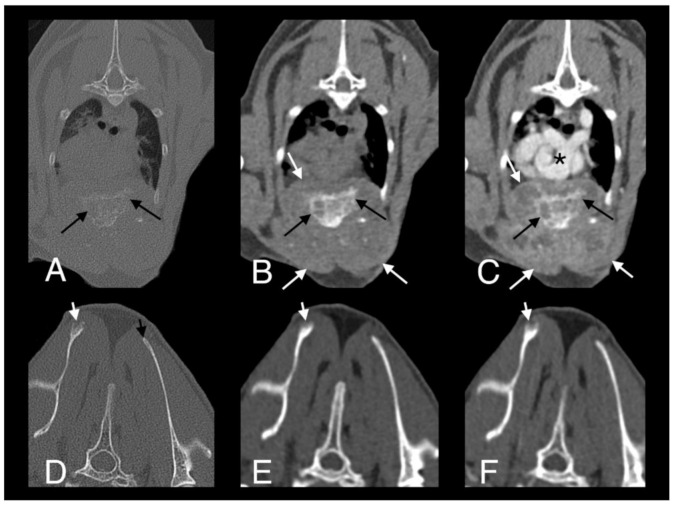
Computed tomography images in transverse plane of cat 2 with different reconstructions: bone (**A**,**D**), soft tissue (**B**,**E**), and soft tissue after contrast medium administration (**C**,**F**). Images **A**–**C** are at the level of the 4th and 5th sternebrae. There is a large, poorly marginated, partially mineralized and heterogeneously contrast enhancing mass infiltrating the adjacent pectoral musculature (white long arrows). Note severe secondary osteolysis of the sternebrae (black long arrows). Marked dorsal displacement of the heart is also visible (asterisk). Images **D**–**F** are at the level of the scapulae. Along the dorsal margin of the right scapula a subtle lytic lesion and mild contrast enhancement in the adjacent soft tissues are visible (short white arrows). The dorsal margin of the left scapula is normal (short black arrow).

A full-body CT scan was performed. A large, poorly marginated, partially mineralized, and heterogeneously contrast-enhancing mass was identified along the pectoral region between the thoracic inlet and the sixth sternebra. Severe secondary osteolysis of sternebrae four and five was evident. The mass infiltrated the superficial and pectoral muscles bilaterally and extended dorsally within the thoracic cavity causing marked dorsal displacement of the heart and mild dorsal displacement of both cranial lung lobes (Figure 8A–C). A subtle lytic lesion was identified along the dorsal margin of the right scapula, with mildly contrast enhancing adjacent soft tissues (Figure 8D–F). Numerous well-marginated nodular soft tissue attenuating lesions, with associated heterogeneous contrast enhancement, were visible within the subcutaneous tissues of the ventral and dorsal thoracic wall. A generalized bronchial pattern was evident, with ill-defined areas of increased pulmonary attenuation, which was more pronounced in the cranial lung lobes. There was also regional lymphadenopathy.

A punch biopsy sample was obtained from one of the skin lesions and sent for PCR to Leeds Teaching Hospitals, as for cat 1. An IGRA was run concurrently; as for cat 1, there was no response to the ESAT-6/CFP-10 cocktail; however, unlike cat 1, the responses to PPDA and PPDB were equivalent and the result was interpreted as infection with a member of the MTBC, most likely *M. microti*. The PCR results (using the same commercial kit followed by 16S rRNA sequencing as for cat 1) were obtained following the TB diagnosis by IGRA, which identified mycobacterial DNA and sequenced as *M. kansasii*. Treatment with rifampicin (8.75 mg/kg PO q24 h; Bova), azithromycin (7.5 mg/kg PO q24 h; Bova) and liquid pradofloxacin (7.5 mg/kg PO q24 h; Veraflox, Bayer) was initiated. Three weeks later, the patient’s skin lesions had started to improve. Repeat hematology and serum biochemistry, for monitoring possible side effects of the antibiotic therapy, was unremarkable.

**Figure 9:**
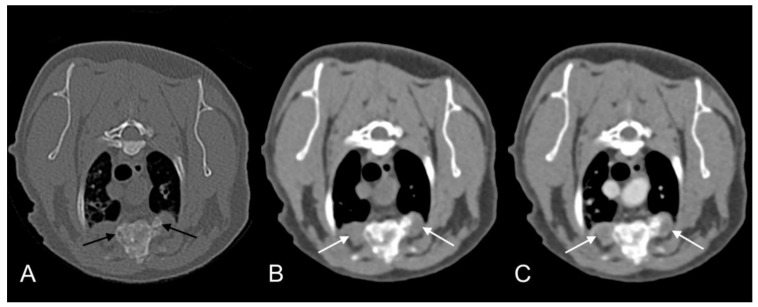
Computed tomography images in transverse plane of cat 2 with different reconstructions: bone (**A**), soft tissue (**B**), and soft tissue after the administration of contrast medium (**C**). Images are at the level of the 4th and 5th sternebrae. Note moderate remodeling of the sternebrae with much more solid bone and no new osteolytic or proliferative changes (black arrows). The soft tissue component of the lesion within the pectoral muscles is markedly reduced in size and exhibits less contrast uptake (white arrows). Compare to Figure 8, images were taken at the same level.

Monitoring has continued. Four weeks later, the patient’s skin lesions had continued to improve. Repeated hematology and serum biochemistry were performed and there were no changes suggestive of hepatotoxicity secondary to rifampicin therapy, or neutropenia or thrombocytopenia secondary to pradofloxacin therapy. Fourteen weeks after starting triple antibiotic therapy, the skin lesions were almost completely healed and on a repeated full-body CT scan there was mild regression of the aggressive soft tissue mass infiltrating the pectoral muscles and resolution of the lytic lesion in the right scapula. However, there was only very mild improvement in the broncho-interstitial lung pattern. Daily treatment with rifampicin and azithromycin was continued but, on alternate days, the doses were doubled to 17.5 mg/kg PO and 15 mg/kg PO, respectively. Pradofloxacin was continued at the same dose and interval as before. A total of ten weeks later (24 weeks after starting treatment), the patient was tolerating the increased dose of rifampicin and azithromycin, and full-body CT showed further regression of the sternebral mass and moderate regression of the bronchial lung pattern. N-acetyl cysteine (NAC) (600 mg/cat PO q12 h; Essential Healthcare) was added to the treatment protocol to try to speed resolution due to its direct antimycobacterial effects as well as hepatoprotective activity [52,53]. The patient presented 21 weeks later (45 weeks after starting treatment) for repeated examinations. Hematology and serum biochemistry findings were unremarkable and full-body CT scan showed further improvement of the size and appearance of the sternebral mass and almost complete resolution of the pulmonary changes (Figure 9). There was moderate remodeling of the sternebrae, with much more solid bone present and no new osteolytic or proliferative changes. The soft tissue component of the lesion within the pectoral muscles was also markedly reduced in size and exhibited less contrast uptake. Antimicrobial therapy was ceased at this point, in part due to the cat becoming progressively reluctant to take oral medication. Careful monitoring of the patient has continued and there has been no evidence of relapse of active disease; a repeat CT scan three and a half months after stopping treatment showed minimal further change in the appearance of the lesions.

## 3. Discussion

This case report describes a rare infection with *M. kansasii* in two sibling cats from the same indoor-only household that developed clinical signs two and a half years apart from each other. Both cats had osteolytic and pulmonary pathology attributable to mycobacterial disease. Cutaneous lesions were also seen in both cats, one of which was confirmed as mycobacterial in origin (cat 2). Mycobacteria were not seen in the other cat’s (cat 1) tail lesion and although not fully investigated, self-trauma due to neuropathic pain from the spinal lesion is a possible cause. Infection with *M. kansasii* was determined by PCR on a FNA of a lumbosacral mass in cat 1 and cutaneous punch biopsy in cat 2; however, the IGRA was discordant, inferring *M. avium*-complex and *M. tuberculosis*-complex infection, respectively. Both cats made a full recovery following antimicrobial therapy with rifampicin, azithromycin, and pradofloxacin, although this took eight months in cat 1, and 11 months in cat 2 (which also received additional N-acetyl cysteine). This report shows that while systemic *M. kansasii* mycobacteriosis in the cat can be challenging to diagnose and treat, successful resolution can be achieved.

Somewhat uniquely among NTM, *M. kansasii* has been frequently isolated from supposedly potable safe drinking water [20,54,55], rather than from the soil or natural water sources [20]. While epidemiological evidence suggests that some human infections may originate from contaminated tap water [56], clinical isolates often form a genetically distinct population compared to *M. kansasii* isolates originating from potable water sources spatiotemporally related, suggesting an alternative source of infection [57]. Additionally, aerogenic infection is perhaps more likely, given their pulmonary pattern of disease [12]. Human-to-human transmission is not thought to occur, although cases of familial clustering have been identified [58,59]. 

The source, route, and dynamics of infection in these two cats remain unknown; both cats were rescued from a multi cat household in Italy, and then lived in Philadelphia and New York, USA, prior to moving to Scotland in 2014. When in Italy, as kittens, they had an outdoor feral lifestyle and had evidence of a poor start in life, demonstrated by the retinal changes suggestive of historical taurine deficiency in cat 1. While it is not possible to learn more about their early life, the kittens could have been infected at that time, from either water, aerosol, food, or cutaneous contamination. After adoption, they have been kept as strictly indoor-only cats, with no known access to rodents or other wildlife species, there have been no other pets in the household, and neither cat had been fed a raw food diet [60]. *M. kansasii* has been infrequently cultured from human cases of mycobacteriosis in Scotland [61], demonstrating a local burden of infection. It is therefore possible that these cats became infected after relocating to Scotland, although how this could have occurred is unclear. One possibility is that, as for cases of human *M. kansasii* disease, contaminated tap water could have been the source of infection; however, there have been no signs of ill health reported in either of the of the adult owners nor their infant. The water supply has not been tested. Another case report of *M. kansasii* infection in an indoor cat also failed to demonstrate the route of infection [33]. 

On consideration, cutaneous or aerosol infections appear most likely. Cutaneous lesions were initially reported in both cats (confirmed as mycobacterial infection in cat 2 but not in cat 1), which may suggest primary cutaneous infection. This would be in keeping with most cases of feline mycobacterial disease—MTBC and NTM—which typically present with cutaneous lesions, which may involve local lymph nodes and subsequently spread, putatively via the hematogenous route, to the lungs [1]. Cats are thought to become infected when hunting rodent prey, resulting in wounds at “fight and bite” sites [35]. Infection may also arise from contamination of pre-existing cutaneous abrasions [35]. This may offer an explanation for how these cats became infected, with cutaneous abrasions becoming infected by contaminated water, resulting in a primary cutaneous focus of disease which then spread systemically [1]. However, both cats could also have been infected by inhalation. They both had a singular large pulmonary lesion that had become mineralized, suggesting chronicity of these lesions. Infection may have then disseminated to the skin, peripheral bones and joints, and to the lung parenchyma, with a long period of dormancy from primary infection to the onset of clinical disease.

It is also unknown whether the cats became infected simultaneously from the same source, or separately on two different occasions from the same or different sources, or if one cat transmitted the infection to the other cat. The owners reported the cats to be very close, snuggling up in close contact, and mutually grooming one another, which could present a route of transmission from one cat to the other. Unfortunately, mycobacterial culture was not performed in either of these cases, and unfixed clinical material from the lesions is no longer available; otherwise, whole genome sequencing could be employed to investigate whether the *M. kansasii* isolates from both cats were identical, inferring a single point of infection or possible cat-to-cat transmission. Other molecular techniques such as variable-number tandem repeat and restriction fragment length polymorphism analysis, which have been well established for investigating the epidemiology of MTBC infections [62], are becoming established for other mycobacteria including *M. kansasii* [63]. Samples should be prospectively collected from future cases of this infection to increase our knowledge of the epidemiology and genetic variation of this pathogen. 

Clustering of infection is thought to arise from hereditary predisposition and increased susceptibility, or to reflect a shared contaminated environment [58,59]. Neither of these cats showed clinical signs of immunosuppression; historical testing for FeLV and FIV had been performed after they were adopted from a multi-cat household and prior to moving to Scotland, with both cats testing negative on these two occasions. Rarer causes of genetic immunosuppression have been identified in cats with disseminated NTM disease [64], so an underlying susceptibility to infection cannot be ruled out. If both cats were infected with *M. kansasii* concurrently, individual differences in their immune system, or external factors, such as environmental stressors, could explain why they did not present with clinical disease at the same time. No humans in the household, nor local cats, have shown evidence of *M. kansasii* infection to support the possibility of a contaminated water supply being the source of infection. However, this pathogen should be considered a possible etiological diagnosis for cases of feline mycobacteriosis, especially in Scotland.

Obtaining a diagnosis of *M. kansasii* infection proved challenging and demonstrated some of the limitations of current tests available for mycobacterial disease. An official diagnosis of mycobacterial disease in domestic cats in the UK can only be obtained through specialist culture [65]. However, this was not performed in either of these cases due to the poor sensitivity of this test [1], the delay in obtaining results [40], and the legal framework surrounding companion animal mycobacterial disease being biased towards investigating potential *M. bovis* infections in the UK, which was not suspected in either of these cats as they were living in Scotland, which is recognised as Officially Bovine Tuberculosis Free. More rapid alternative tests were employed. Both cats had lesions consistent with mycobacterial disease on histopathology or cytology, with ZN staining being positive in cat 1, but negative for cat 2. However, the sensitivity of detecting AFB on ZN staining is poor [39], with this being subject to variability between examiners [37]. Therefore, if granulomatous to pyogranulomatous inflammation is identified on histopathology but ZN staining for AFB is negative, as was the case for cat 2, this should still be considered highly suggestive of mycobacterial infection and follow-up investigations should be conducted [37]. Other causes of (pyo)granulomatous dermal lesions in cats are uncommon, but include other bacteria, viruses (e.g., Feline Infectious Peritonitis), parasites (e.g., Toxoplasmosis), fungi or non-infectious causes like foreign bodies [66].

For cat 1, the IGRA showed a PPDA-biased response, whereas for cat 2 the responses to avian and bovine PPD were equivalent; a quantifiable IFNγ response was not detected from either cat in response to stimulation with the ESAT-6/CFP-10 antigenic cocktail. For cat 1 the result was interpreted as MAC infection, whereas for cat 2 the result was taken to indicate infection with *M. microti*; *M. bovis* was considered unlikely because the cat lived in Scotland and there was no history of hunting or feeding a raw food diet [60]. *M. kansasii* (including its former associated subtypes) is one of the few NTM that encode ESAT-6 and CFP-10 [25,67], and studies have shown a high degree of homology between *M. kansasii* and *M. bovis* ESAT-6 and CFP-10, resulting in similar T-cell recognition for these two mycobacterial species [68]. This could compromise the ability to distinguish between *M. kansasii* and other mycobacteria that encode these two genes; humans infected with *M. kansasii* have shown false-positive results for TB i.e., *M. tuberculosis* [69] by tuberculin skin testing and IGRA [70,71]. Neither cat produced sufficient IFNγ to be considered positive for ESAT-6/CFP-10; however, *M. kansasii* appears less potent at stimulating T-lymphocytes to secrete IFNγ than MTBC pathogens in people [71], so this may also be the case with cats. Expression of CFP-10 also appears to be reduced in some environmental isolates of *M. kansasii* subtypes [67], which may result in reduced T-lymphocyte IFNγ production. Previous studies in cats have suggested that a PPDA-biased response may be seen with NTM infections other than MAC [49], which was the pattern of results from cat 1. Where an equivalent PPDA and PPDB response is observed, caution should be exercised when categorising the infection as either NTM (including MAC) or MTBC; in these cases, supportive epidemiological data should be taken into consideration, and the authors recommend performing secondary testing such as PCR or mycobacterial culture. For some owners, a diagnosis of TB, regardless of the underlying pathogen, could result in euthanasia of the cat due to concerns about zoonotic risk. Additionally, misdiagnosing the infecting species of mycobacteria could result in suboptimal treatment being prescribed. 

The veterinary literature describing diagnostic imaging features associated with feline mycobacterial infections is sparse. Previous publications are limited to a retrospective case series describing radiographic changes in 33 cats [72], CT features in 20 cats [73], serial CT scans in 9 cats [74], CT findings in 4 cats with joint-associated TB [75,76], and a relatively small number of single case reports describing the radiographic features of feline mycobacteriosis [76,77]. None of the published articles report infection with *M. kansasii*; however, as the specific mycobacterial species was not known in all the cases, we cannot exclude a possible case of *M. kansasii* in some of those previously published reports. In general, thoracic radiography findings are variable in cats with mycobacteriosis, including tracheobronchial lymphadenopathy, interstitial or miliary lung infiltration, localized lung consolidation, or pleural effusion [72]. Abdominal radiography and ultrasound examination may reveal hepato- or splenomegaly abdominal masses, mineralized mesenteric lymph nodes, or ascites. Neither of our patients showed abdominal changes. Computed tomography provides increased sensitivity for detecting lesions compared to radiography, and a diffuse structured interstitial lung pattern is the most common, being either nodular or reticulo-nodular in nature [73]. Our patients showed lesions consistent with the previous reports, where cat 1 developed a pulmonary mass and cat 2 showed more diffuse pulmonary changes with interstitial infiltration. Soft tissue masses as well as osseous lesions have been reported previously, with the latter tending to consist of areas of lysis and sclerosis, osteoarthritis, discospondylitis, or periostitis [72]. Resolution of osteolytic changes, as observed in both of our patients, has not been previously described, perhaps because amputation of the affected regions has typically been advised. In a series of four cases of mycobacterial arthritis, two underwent treatment and recovered uneventfully following amputation of the affected limb and triple antimicrobial therapy (as given to the two cats in the current report); one case received three months of triple antimicrobial therapy, while the other received two months of triple antimicrobial therapy, followed by treatment with rifampicin and azithromycin for a further four months [75]. The marked improvement in our patients suggests that osteolytic changes may be reversible with appropriate treatment, although amputation may still be recommended, where possible, especially in cases of *M. bovis* infection given the increased risk to human health. The MRI findings of feline mycobacterial infections have not previously been described. The decision to undertake MRI in cat 1 was made given the unusual presentation giving rise to the suspicion of a neurological component to the clinical signs. The CT and MRI complemented one another and both modalities contributed to obtaining an appropriate diagnosis, facilitated sampling and CT served as a monitoring tool in these two cases. 

The perceived zoonotic risk of feline mycobacterial infections is often overstated; in the past 150 years, there have only been six recorded cases of feline-to-human transmission of *M. bovis* [78,79], with the common feature of purulent draining lesions in all of these cases [78]. Zoonotic transmission of the other major causes of feline mycobacteriosis, namely *M. microti* and *M. avium,* has not been identified. Mechanical transmission of *M. kansasii* from animals to humans has been reported rarely [80], so it is theoretically possible for zoonotic transmission from cat-to-human, especially if an individual is immunocompromised, hence the importance of obtaining an accurate diagnosis to inform the risk posed. 

Treating mycobacterial infections in cats can be challenging [37]; however, with appropriate therapy, remission rates exceeding 80% have been reported [8,15]. In human medicine there are well established protocols for treating specific NTM infections [81]. Recommended antimycobacterial therapy for treating *M. kansasii* infection is a combination of rifampin, isoniazid, and ethambutol, similar to protocols for treating TB; however, the duration of therapy is often longer for *M. kansasii,* often lasting 18 months as treatment is recommended for 12 months beyond achieving microbiological resolution [81]. Fluoroquinolones and macrolides are suggested for treating rifampin-resistant strains [81], and are routinely used for treating cases of feline mycobacteriosis due to the toxicities associated with isoniazid and ethambutol [8,82]. Pyrazinamide is not used in the treatment of feline mycobacteriosis as *M. bovis* is a common cause of disease in this species, and it is naturally resistant to this drug [83,84]. As far as the authors are aware, successful resolution of *M. kansasii* infection in veterinary species has only been reported twice, in two Black Bearded Sakis (*Chiropotes satanas*), a type of New World monkey [29], and another indoor cat, which was treated with rifampicin and clarithromycin [33]. The previous lack of success may have resulted from inappropriate selection of antimicrobials [32], or inadequate duration of treatment [30]. 

Treatment protocols can vary depending on which species of mycobacteria is identified [35], and the lack of published data on successful treatment of *M. kansasii* infection in animals limits our knowledge on the optimal antibiotic regimen for this pathogen. A treatment protocol consisting of rifampicin, azithromycin, and pradofloxacin, established by the senior author, has been highly efficacious for treating cases of feline TB [8], and was implemented for treating these two cats given the author’s familiarity with the protocol and awareness of potential side effects. Cat 1 was treated for eight months with high doses of rifampicin, azithromycin, and pradofloxacin, while cat 2 was treated for 11 months with the same three drugs (although the doses of rifampicin and azithromycin were lower) and NAC was added in for the last five months. 

The NAC was added because experimental animal models of *M. tuberculosis* infection, and in vitro studies, have shown NAC to have direct antimycobacterial effects [52,53]. One study investigating the pharmacokinetics of NAC in cats estimated effective in vitro target concentrations could be established after oral dosing with 100 mg/kg every 12 or 24 h [85]; however, the rapid total body clearance and short half-life of NAC may result in concentrations that are less efficacious against mycobacteria in vivo. When NAC was given for 30 or 60 days to guinea pigs infected via aerosol with *M. tuberculosis* at a dose of 400 mg/kg, the number of bacteria present within the spleen was lower compared to control animals; however, there was no difference in bacterial numbers recovered from the lungs or peribronchial lymph nodes [52]. A mouse model showed a reduction in bacterial numbers recovered from the lungs following a seven-day course of NAC (400 mg/kg) following intratracheal administration of *M. tuberculosis* [53]. However, differences in the routes of infection, the pathogen studied and the host immune response may not accurately represent what occurs in naturally infected cats. Addition of NAC to TB protocols in humans, at a dose of 1200 mg/day, showed an increase in the number of sputum samples converting to negative after three weeks compared to those receiving placebo [86], suggesting NAC may have some benefits as an add-on for treating mycobacterial infections. In our report, cat 2 received a dose of 136 mg/kg every 24 h, which may have conferred some direct antimycobacterial activity; further studies are needed to evaluate the efficacy of NAC as an adjuvant treatment for feline mycobacterial infections. 

Both patients also received supportive therapy as appropriate i.e., hepatoprotectants (SAMe, silybin, and for cat 2 additional NAC [53,87,88]) and analgesia, plus topical fusidic acid for cat 1. 

Higher starting doses of rifampicin and azithromycin in cat 1 were selected given the extent of the infection and the bony involvement (DGM, unpublished data). Retrospectively, higher doses may have reduced the duration of disease in cat 2. The lower doses were dictated by the new formulation of rifampicin in the UK, which is compounded with azithromycin (rifampicin 35 mg and azithromycin 30 mg, per capsule; Bova), plus difficulties in medicating this cat which suggested starting with a single capsule daily. The reformulated capsules improve cat compliance, but meant that cat 2 could receive either 8.75 mg/kg of rifampicin daily, or twice daily, with the latter raising concerns about toxicity as the recommended daily dose is 10 mg/kg. When the treatment response was limited after 14 weeks, the dose was increased, with a single capsule being given one day, and two the next, alternately (i.e., 13 mg/kg over the two days). No clinical or hematological signs of hepatotoxicity were noted in either patient; however, cat 1 developed neutropenia, a recognised side effect of high, prolonged doses of pradofloxacin, where myelosuppression has been recorded in dogs [89]. Follow-up investigations have revealed no recurrence of clinical signs in cat 1 for over two and a half years since ceasing antimycobacterial therapy (at the time of writing), showing that *M. kansasii* can be successfully treated in the domestic cat. Cat 2 had a repeated CT scan three and a half months after stopping antimicrobial therapy, with no significant changes in the appearance of lesions being seen. This cat is still being monitored closely.

## 4. Conclusions

This case report describes the clinical presentation, diagnostic evaluation and successful treatment of two sibling cats with systemic *M. kansasii* infection. Clinical resolution was achieved after 8 and 11 months of triple antibiotic therapy, respectively, and, while rare, *M. kansasii* should be considered an opportunistic pathogen in cats that can be mistaken for TB and can be challenging to diagnose and treat. This is also the first case reported of familial clustering of *M. kansasii* infection in this species.

## Note

These cases were both seen at the RDSVS when all authors were working there.

## References

[1] Gunn-Moore D.A., McFarland S.E., Brewer J.I., Crawshaw T.R., Clifton-Hadley R.S., Kovalik M., Shaw D.J. (2011). Mycobacterial disease in cats in Great Britain: I. Culture results, geographical distribution and clinical presentation of 339 cases. J. Feline Med. Surg..

[2] Rüfenacht S., Bögli-Stuber K., Bodmer T., Jaunin V.F., Jmaa D.C., Gunn-Moore D.A. (2011). *Mycobacterium microti* infection in the cat: A case report, literature review and recent clinical experience. J. Feline Med. Surg..

[3] Laprie C., Duboy J., Malik R., Fyfe J. (2013). Feline cutaneous mycobacteriosis: A review of clinical, pathological and molecular characterization of one case of *Mycobacterium microti* skin infection and nine cases of feline leprosy syndrome from France and New Caledonia. Vet. Dermatol..

[4] Michelet L., de Cruz K., Zanella G., Aaziz R., Bulach T., Karoui C., Hénault S., Joncour G., Boschiroli M.L. (2015). Infection with *Mycobacterium microti* in animals in France. J. Clin. Microbiol..

[5] Černá P., O’Halloran C., Sjatkovskaj O., Gunn-Moore D.A. (2019). Outbreak of Tuberculosis caused by *Mycobacterium bovis* in a cattery of Abyssinian cats in Italy. Transbound. Emerg. Dis..

[6] O’Brien C.R., Malik R., Globan M., Reppas G., McCowan C., Fyfe J.A. (2017). Feline leprosy due to *Candidatus* ‘Mycobacterium tarwinense’: Further clinical and molecular characterisation of 15 previously reported cases and an additional 27 cases. J. Feline Med. Surg..

[7] Davies J.L., Sibley J.A., Myers S., Clark E.G., Appleyard G.D. (2006). Histological and genotypical characterization of feline cutaneous mycobacteriosis: A retrospective study of formalin-fixed paraffin-embedded tissues. Vet. Dermatol..

[8] O’Halloran C., Gunn-Moore D. (2017). Mycobacteria in cats: An update. Practice.

[9] Burthe S., Bennett M., Kipar A., Lambin X., Smith A., Telfer S., Begon M. (2008). Tuberculosis (*Mycobacterium microti*) in wild field vole populations. Parasitology.

[10] List of Prokaryotic names with Standing in Nomenclature. Search: Mycobacterium. http://lpsn.dsmz.de/search?word=mycobacterium.

[11] Whittington R., Donat K., Weber M., Kelton D., Nielsen S.S., Eisenberg S., Arrigoni N., Juste R., Saez J., Dhand N. (2019). Control of paratuberculosis: Who, why and how. A review of 48 countries. BMC Vet. Res..

[12] Primm T., Lucero C.A., Falkinham J.O. (2004). Health Impacts of Environmental Mycobacteria. Clin. Microbiol. Rev..

[13] Runyon E.H. (1959). Anonymous Mycobacteria in Pulmonary Disease. Med. Clin. N. Am..

[14] Scollard D.M., Dacso M.M., Abad-Venida M.L. (2015). Tuberculosis and Leprosy: Classical Granulomatous Diseases in the Twenty-First Century. Dermatol. Clin..

[15] O’Brien C.R., Malik R., Globan M., Reppas G., McCowan C., Fyfe J.A. (2017). Feline leprosy due to *Mycobacterium lepraemurium*: Further clinical and molecular characterisation of 23 previously reported cases and an additional 42 cases. J. Feline Med. Surg..

[16] Appleyard G.D., Clark E.G. (2002). Histologic and Genotypic Characterization of a Novel Mycobacterium Species Found in Three Cats. J. Clin. Microbiol..

[17] O’Brien C.R., Malik R., Globan M., Reppas G., McCowan C., Fyfe J.A. (2017). Feline leprosy due to *Candidatus* ‘Mycobacterium lepraefelis’: Further clinical and molecular characterisation of eight previously reported cases and an additional 30 cases. J. Feline Med. Surg..

[18] Vincent V., Gutierres M. (2007). Mycobacterium: Laboratory characteristics of slowly growing mycobacteria. Manual of Clinical Microbiology.

[19] Buhler V.B., Pollak A. (1953). Human Infection with Atypical Acid-Fast Organisms; Report of Two Cases with Pathologic Findings. Am. J. Clin. Pathol..

[20] Falkinham III J.O. (1996). Epidemiology of infection by nontuberculous mycobacteria. Clin. Microbiol. Rev..

[21] Johnston J.C., Chiang L., Elwood K. (2017). Mycobacterium kansasii. Microbiol. Spectr..

[22] Han S.H., Kim K.M., Chin B.S., Choi S.H., Lee H.S., Kim M.S., Jeong S.J., Choi H.K., Kim C.O., Choi J.Y. (2010). Disseminated *Mycobacterium kansasii* Infection Associated with Skin Lesions: A Case Report and Comprehensive Review of the Literature. J. Korean Med. Sci..

[23] Bercovier H., Vincent V. (2001). Mycobacterial infections in domestic and wild animals due to *Mycobacterium marinum, Mycobacterium fortuitum, Mycobacterium chelonae, Mycobacterium porcinum, Mycobacterium farcinogenes, Mycobacterium smegmatis, Mycobacterium scrofulaceum, Mycobacterium xenopi, Mycobacterium kansasii, Mycobacterium simiae* and *Mycobacterium genavense*. Rev. Sci. Tech..

[24] Thacker T.C., Robbe-Austerman S., Harris B., Palmer M.V., Waters W.R. (2013). Isolation of mycobacteria from clinical samples collected in the United States from 2004 to 2011. BMC Vet. Res..

[25] Jagielski T., Borówka P., Bakuła Z., Lach J., Marciniak B., Brzostek A., Dziadek J., Dziurzyński M., Pennings L., van Ingen J. (2020). Genomic Insights into the *Mycobacterium kansasii* Complex: An Update. Front. Microbiol..

[26] Hall P.B., Bender L.C., Garner M.M. (2005). Mycobacteriosis in a Black-Tailed Deer (*Odocoileus hemionus columbianus*) Caused by *Mycobacterium kansasii*. J. Zoo Wildl. Med..

[27] Braun U., Previtali M., Gaustchi A., Forster E., Steininger K., Irmer M., Reichle S., Sydler T., Widerkehr D., Ruetten M. (2009). Sonographic findings in an alpaca with *Mycobacterium kansasii* infection. Schweiz. Arch. Tierheilkd..

[28] Miller M., Terrell S., Lyashchenko K., Greenwald R., Harris B., Thomsen B.V., Fontenot D., Stetter M., Neiffer D., Fleming G. (2011). *Mycobacterium kansasii* Infection in a Bontebok (*Damaliscus pygaragus dorcas*) Herd: Diagnostic Challengens in Differentiating from the *Mycobacterium tuberculosis* Complex. J. Zoo Wildl. Med..

[29] Murai A., Murakami T., Inoue M., Ueda H., Shiihara S., Kimura J., Hirata A., Sakai H., Yanai T. (2012). Pathological Features of *Mycobacterium kansasii* Infection in Black Bearded Sakis (*Chiropotes satanas*). J. Comp. Pathol..

[30] Murai A., Maruyama S., Nagata M., Yuki M. (2013). Mastitis caused by *Mycobacterium kansasii* infection in a dog. Vet. Clin. Pathol..

[31] Schafbuch R., Tinkler S., Lim C.K., Wolking R., Ramos-Vara J. (2018). Disseminated mycobacteriosis caused by *Mycobacterium kansasii* in a pot-bellied pig. J. Vet. Diagn. Investig..

[32] Lee S.H., Go D.M., Woo S.H., Park H.T., Kim E., Yoo H.S., Kim D.Y. (2017). Systemic *Mycobacterium kansasii* Infection in a Domestic Shorthair Cat. J. Comp. Pathol..

[33] Fukano H., Terazono T., Hirabayashi A., Yoshida M., Suzuki M., Wada S., Ishii N., Hoshino Y. (2020). Human Pathogenic *Mycobacterium Kansasii* (Former Subtype I) with Zoonotic Potential Isolated from a Diseased Indoor Pet Cat, Japan.

[34] Foley J.E., Gross T.L., Drazenovich N., Ramiro-Ibanez F., Anacleto E. (2004). Clinical, pathological, and molecular characterization of feline leprosy syndrome in the western USA. Vet. Dermatol..

[35] Gunn-Moore D.A. (2014). Feline mycobacterial infections. Vet. J..

[36] Lalor S.M., Mellanby R.J., Friend E.J., Bowlt K.L., Berry J., Gunn-Moore D. (2012). Domesticated Cats with Active Mycobacteria Infections have Low Serum Vitamin D (25(OH)D) Concentrations. Transbound. Emerg. Dis..

[37] Gunn-Moore D.A., McFarland S.E., Schock A., Brewer J.I., Crawshaw T.R., Clifton-Hadley R.S., Shaw D.J. (2011). Mycobacterial disease in a population of 339 cats in Great Britain: II. Histopathology of 225 cases, and treatment and outcome of 184 cases. J. Feline Med. Surg..

[38] Raskin R.E., Raskin R.E., Meyer D.J. (2016). Skin and Subcutaneous Tissue. Canine and Feline Cytology: A Color Atlas and Interpretation Guide.

[39] Gunn-Moore D.A., Gaunt C., Shaw D.J. (2013). Incidence of mycobacterial infections in cats in Great Britain: Estimate from feline tissue samples submitted to diagnostic laboratories. Transbound. Emerg. Dis..

[40] Smith N.H., Crawshaw T., Parry J., Birtles R.J. (2009). *Mycobacterium microti*: More Diverse than Previously Thought. J. Clin. Microbiol..

[41] Aranaz A., Liébana E., Pickering X., Novoa C., Mateos A., Domínguez L. (1996). Use of polymerase chain reaction in the diagnosis of tuberculosis in cats and dogs. Vet. Rec..

[42] Malik R., Hughes M.S., James G., Martin P., Wigney D.I., Canfield P.J., Chen S.C.A., Mitchell D.H., Love D.N. (2002). Feline Leprosy: Two Different Clinical Syndromes. J. Feline Med. Surg..

[43] Fyfe J.A., McCowan C., O’Brien C.R., Globan M., Birch C., Revill P., Barrs V.R.D., Wayne J., Hughes M.S., Holloway S. (2008). Molecular Characterization of a Novel Fastidious Mycobacterium Causing Lepromatous Lesions of the Skin, Subcutis, Cornea, and Conjunctiva of Cats Living in Victoria, Australia. J. Clin. Microbiol..

[44] Reppas G., Fyfe J., Foster S., Smits B., Martin P., Jardine J., Lam A., O’Brien C., Malik R. (2013). Detection and identification of mycobacteria in fixed stained smears and formalin-fixed paraffin-embedded tissues using PCR. J. Small Anim. Pract..

[45] Rhodes S.G., Gruffydd-Jones T., Gunn-Moore D., Jahans K. (2008). Interferon-γ test for feline tuberculosis. Vet. Rec..

[46] Rhodes S.G., Gruffydd-Jones T., Gunn-Moore D., Jahans K. (2008). Adaptation of IFN-gamma ELISA and ELISPOT tests for feline tuberculosis. Vet. Immunol. Immunopathol..

[47] Wood P.R., Jones S.L. (2001). BOVIGAM: An in vitro cellular diagnostic test for bovine tuberculosis. Tuberculosis.

[48] Pai M., Denkinger C.M., Kik S.V., Rangaka M.X., Zwerling A., Oxlade O., Metcalfe J.Z., Cattamanchi A., Dowdy D.W., Dheda K. (2014). Gamma Interferon Release Assays for Detection of *Mycobacterium tuberculosis* Infection. Clin. Microbiol. Rev..

[49] Rhodes S.G., Gunn-Moore D., Boschiroli M.L., Schiller I., Esfandiari J., Greenwald R., Lyashchenko K.P. (2011). Comparative study of IFNγ and antibody tests for feline tuberculosis. Vet. Immunol. Immunopathol..

[50] Kirscher P., Springer B., Vogel U., Meier A., Wrede A., Kiekenbeck M., Bange F.C., Böttger E.C. (1993). Genotypic identification of mycobacteria by nucleic acid sequence determination: Report of a 2-year experience in a clinical laboratory. J. Clin. Microbiol..

[51] Richter E., Rüsch-Gerdes S., Hillemann D. (2006). Evaluation of the GenoType Mycobacterium Assay for Identification of Mycobacterial Species from Cultures. J. Clin. Microbiol..

[52] Palanisamy G.S., Kirk N.M., Ackart D.F., Shanley C.A., Orme I.M., Basaraba R.J. (2011). Evidence for Oxidative Stress and Defective Antioxidant Response in Guinea Pigs with Tuberculosis. PLoS ONE.

[53] Amaral E.P., Conceição E.L., Costa D.L., Rocha M.S., Marinho J.M., Cordeiro-Santos M., D’Império-Lima M.R., Barbosa T., Sher A., Andrade B.B. (2016). N-acetyl-cysteine exhibits potent anti-mycobacterial activity in addition to its known anti-oxidative functions. BMC Microbiol..

[54] Steadham J.E. (1980). High-catalase strains of *Mycobacterium kansasii* isolated from water in Texas. J. Clin. Microbiol..

[55] Engel H.W., Berwald L.G., Havelaar A.H. (1980). The occurrence of *Mycobacterium kansasii* in tapwater. Tubercle.

[56] Engel H.W., Berwald L.G., Lindeboom B.W., Havelaar A.H. (1981). *Mycobacterium kansasii* infections in the Netherlands: A brief summary. Rev. Infect. Dis..

[57] Thomson R., Tolson C., Huygens F., Hargreaves M. (2014). Strain variation amongst clinical and potable water isolates of *M. kansasii* using automated repetitive unit PCR. Int. J. Med. Microbiol..

[58] Penny M.E., Cole R.B., Gray J. (1982). Two cases of *Mycobacterium kansasii* infection occurring in the same household. Tubercle.

[59] Colombo R.E., Hill S.C., Claypool R.J., Holland S.M., Olivier K.N. (2010). Familial clustering of pulmonary nontuberculous mycobacterial disease. Thorax.

[60] O’Halloran C., Ioannidi O., Reed N., Murtagh K., Dettemering E., Van Poucke S., Gale J., Vickers J., Burr P., Gascoyne-Binzi D. (2019). Tuberculosis due to *Mycobacterium bovis* in pet cats associated with feeding a commercial raw food diet. J. Feline Med. Surg..

[61] Russell C.D., Claxton P., Doig C., Seagar A.-L., Rayner A., Laurenson I.F. (2014). Non-tuberculous mycobacteria: A retrospective review of Scottish isolates from 2000 to 2010. Thorax.

[62] Cowan L.S., Mosher L., Diem L., Massey J.P., Crawford J.T. (2002). Variable-Number Tandem Repeat Typing of *Mycobacterium tuberculosis* Isolates with Low Copy Numbers of IS*6110* by Using Mycobacterial Interspersed Repetitive Units. J. Clin. Microbiol..

[63] Bakuła Z., Brzostek A., Borówka P., Żaczek A., Szulc-Kiełbik I., Podpora A., Parniewski P., Strapagiel D., Dziadek J., Proboszcz M. (2018). Molecular typing of *Mycobacterium kansasii* using pulsed-field gel electrophoresis and a newly designed variable-number tandem repeat analysis. Sci. Rep..

[64] Meeks C., Levy J.K., Crawford P.C., Farina L.L., Origgi F., Alleman R., Seddon O.M., Salcedo A., Hirsch B.J., Hirsch S.G. (2008). Chronic Disseminated *Mycobacterium xenopi* Infection in a Cat with Idiopathic CD4+ T Lymphocytopenia. J. Vet. Intern. Med..

[65] Middlemiss C., Clark J. (2018). *Mycobacterium* in pets. Vet. Rec..

[66] Gross T.L., Ihrke P.J., Walder E.J., Affolter V.K. (2005). Infectious nodular and diffuse granulomatous and pyogranulomatous diseases of the dermis. Skin Diseases of the Dog and Cat: Clinical and Histopathologic Diagnosis.

[67] Arend S.M., de Haas P., Leyten E., Rosenkrands I., Rigouts L., Andersen P., Mijs W., van Dissel J.T., van Soolingen D. (2005). ESAT-6 and CFP-10 in Clinical versus Environmental Isolates of *Mycobacterium kansasii*. J. Infect. Dis..

[68] Vordermeier H.M., Brown J., Cockle P.J., Franken W.P.J., Arend S.M., Ottenhoff T.H.M., Jahans K., Hewinson R.G. (2007). Assessment of Cross-Reactivity between *Mycobacterium bovis* and *M. kansasii* ESAT-6 and CFP-10 at the T-Cell Epitope Level. Clin. Vaccine Immunol..

[69] Arend S.M., van Meijgaarden K.E., de Boer K., Cerda de Palou E., van Soolingen D., Ottenhoff T.H.M., van Dissel J.T. (2002). Tuberculin skin testing and in vitro T cell responses to ESAT-6 and culture filtrate protein 10 after infection with *Mycobacterium marinum* or *M. kansasii*. J. Infect. Dis..

[70] Kuznetcova T., Sauty A., Herbort C. (2012). Uveitis with occult choroiditis due to *Mycobacterium kansasii*: Limitations of interferon-gamma release assay (IGRA) tests (case report and mini-review on ocular non-tuberculous mycobacteria and IGRA cross-reactivity). Int. Ophthalmol..

[71] Sato R., Nagai H., Matsui H., Kawabe Y., Takeda K., Kawashima M., Suzuki J., Ohshima N., Masuda K., Yamane A. (2016). Interferon-gamma release assays in patients with *Mycobacterium kansasii* pulmonary infection: A retrospective survey. J. Infect..

[72] Bennett A.D., Lalor S., Schwarz T., Gunn-Moore D.A. (2011). Radiographic findings in cats with mycobacterial infections. J. Feline Med. Surg..

[73] Major A., Holmes A., Warren-Smith C., Lalor S., Littler R., Schwarz T., Gunn-Moore D. (2016). Computed tomographic findings in cats with mycobacterial infection. J. Feline Med. Surg..

[74] Major A., O’Halloran C., Holmes A., Lalor S., Littler R., Spence S., Schwarz T., Gunn-Moore D. (2018). Use of computed tomography imaging during long-term follow-up on nine feline tuberculosis cases. J. Feline Med. Surg..

[75] Lalor S.M., Clark S., Pink J., Parry A., Scurrell E., Fitzpatrick N., Watson F., O’Halloran C., Gunn-Moore D. (2017). Tuberculosis joint infections in four domestic cats. JFMS Open Rep..

[76] Foster S.F., Martin P., Davis W., Allan G.S., Mitchell D.H., Malik R. (1999). Chronic pneumonia caused by *Mycobacterium thermoresistibile* in a cat. J. Small Anim. Pract..

[77] Baral R.M., Metcalfe S.S., Krockenberger M.B., Catt M.J., Barrs V.R., McWhirter C., Hutson C.A., Wigney D.I., Martin P., Chen S.C.A. (2006). Disseminated *Mycobacterium avium* infection in young cats: Overrepresentation of Abyssinian cats. J. Feline Med. Surg..

[78] Gunn-Moore D.A., Lalor S., Mukundan H., Chambers M.A., Waters W.R., Larsen M.H. (2015). Tuberculosis in Companion Animal Species. Tuberculosis, Leprosy and Mycobacterial Diseases of Man and Animals: The Many Hosts of Mycobacteria.

[79] O’Connor C.M., Abid M., Walsh A.L., Behbod B., Roberts T., Booth L.V., Thomas H.L., Smith N.H., Palkopoulou E., Dale J. (2019). Cat-to-Human Transmission of *Mycobacterium bovis*, United Kingdom. Emerg. Infect. Dis..

[80] Southern P.M. (2004). Tenosynovitis Caused by *Mycobacterium kansasii* Associated with a Dog Bite. Am. J. Med. Sci..

[81] Griffith D.E., Aksamit T., Brown-Elliott B.A., Catanzaro A., Daley C., Gordin F., Holland S.M., Horsburgh R., Huitt G., Iademarco M.F. (2007). An official ATS/IDSA statement: Diagnosis, treatment, and prevention of nontuberculous mycobacterial diseases. Am. J. Respir. Crit. Care Med..

[82] Gunn-Moore D.A., Jenkins P.A., Lucke V.M. (1996). Feline tuberculosis: A literature review and discussion of 19 cases caused by an unusual mycobacterial variant. Vet. Rec..

[83] Konno K., Feldmann F.M., McDermott W. (1967). Pyrazinamide Susceptibility and Amidase Activity of Tubercle Bacilli. Am. Rev. Respir. Dis..

[84] Sreevatsan S., Pan X., Zhang Y., Kreiswirth B.N., Musser J.M. (1997). Mutations associated with pyrazinamide resistance in *pncA* of *Mycobacterium tuberculosis* complex organisms. Antimicrob. Agents Chemother..

[85] Buur J.L., Diniz P.P.V.P., Roderick K.V., KuKanich B., Tegzes J.H. (2013). Pharmacokinetics of N-acetylcysteine after oral and intravenous administration to healthy cats. Am. J. Vet. Res..

[86] Mahakalkar S.M., Nagrale D., Gaur S., Urade C., Murhar B., Turankar A. (2017). N-acetylcysteine as an add-on to Directly Observed Therapy Short-I therapy in fresh pulmonary tuberculosis patients: A randomized, placebo-controlled, double-blind study. Perspect. Clin. Res..

[87] Martinez-Chantar M.L., Garcia-Trevijano E.R., Latasa M.U., Perez-Mato I., Del Pino M.M.S., Corrales F.J., Avila M.A., Mato J.M. (2002). Importance of a deficiency in S-adenosyl-L-methionine synthesis in the pathogenesis of liver injury. Am. J. Clin. Nutr..

[88] Center S.A., Randolph J.F., Warner K.L., McCabe-McClelland J., Foureman P., Hoffmann W.E., Erb H.N. (2005). The effects of S-adenosylmethionine on clinical pathology and redox potential in the red blood cell, liver, and bile of clinically normal cats. J. Vet. Intern. Med..

[89] Sykes J.E., Papich M.G., Sykes J.E. (2014). Antibacterial Drugs. Canine and Feline Infectious Diseases.

